# Mesenchymal Stem Cells Promote Metastasis of Lung Cancer Cells by Downregulating Systemic Antitumor Immune Response

**DOI:** 10.1155/2017/6294717

**Published:** 2017-07-16

**Authors:** Marina Gazdic, Bojana Simovic Markovic, Nemanja Jovicic, Maja Misirkic-Marjanovic, Valentin Djonov, Vladimir Jakovljevic, Nebojsa Arsenijevic, Miodrag L. Lukic, Vladislav Volarevic

**Affiliations:** ^1^Department of Genetics, Faculty of Medical Sciences, University of Kragujevac, 34000 Kragujevac, Serbia; ^2^Department of Microbiology and Immunology, Center for Molecular Medicine and Stem Cell Research, Faculty of Medical Sciences, University of Kragujevac, 69 Svetozar Markovic Street, 34000 Kragujevac, Serbia; ^3^Department of Histology and Embryology, Faculty of Medical Sciences, University of Kragujevac, 34000 Kragujevac, Serbia; ^4^Institute of Microbiology and Immunology, School of Medicine, University of Belgrade, 11000 Belgrade, Serbia; ^5^Institute of Anatomy, University of Bern, 3000 Bern 9, Switzerland; ^6^Department of Physiology, Faculty of Medical Sciences, University of Kragujevac, 34000 Kragujevac, Serbia

## Abstract

Since majority of systemically administered mesenchymal stem cells (MSCs) become entrapped within the lungs, we used metastatic model of lung cancer, induced by intravenous injection of Lewis lung cancer 1 (LLC1) cells, to investigate the molecular mechanisms involved in MSC-mediated modulation of metastasis. MSCs significantly augmented lung cancer metastasis, attenuate concentrations of proinflammatory cytokines (TNF-*α*, IL-17), and increase levels of immunosuppressive IL-10, nitric oxide, and kynurenine in sera of LLC1-treated mice. MSCs profoundly reduced infiltration of macrophages, TNF-*α*-producing dendritic cells (DCs), TNF-*α*-, and IL-17-producing CD4+ T cells but increased IL-10-producing CD4+ T lymphocytes in the lungs of tumor-bearing animals. The total number of lung-infiltrated, cytotoxic FasL, perforin-expressing, TNF-*α*-, and IL-17-producing CD8+ T lymphocytes, and NKG2D-expressing natural killer (NK) cells was significantly reduced in LLC1 + MSC-treated mice. Cytotoxicity of NK cells was suppressed by MSC-conditioned medium. This phenomenon was abrogated by the inhibitors of inducible nitric oxide synthase (iNOS) and indoleamine 2,3-dioxygenase (IDO), suggesting the importance of iNOS and IDO for MSC-mediated suppression of antitumor cytotoxicity of NK cells. This study provides the evidence that MSCs promote lung cancer metastasis by suppressing antitumor immune response raising concerns regarding safety of MSC-based therapy in patients who have genetic susceptibility for malignant diseases.

## 1. Introduction

Mesenchymal stem cells (MSCs) are self-renewable adult stem cells with fibroblast-like morphology that can be found in almost all postnatal tissues [1]. MSCs are currently used in broad number of clinical trials due to their multilineage differentiation potential and immunomodulatory characteristics. MSCs differentiate into the cells of mesodermal origin in vitro and in vivo, but recently published data suggest that under specific culture conditions, plasticity of MSCs should be extended to nonmesenchymal lineages of neuroectodermal (neurons, astrocytes, and oligodendrocytes) or endodermal (hepatocytes) origin [2].

MSCs may promote angiogenesis by transdifferentiation into endothelial cells and through the production of several proangiogenic factors (hepatocyte growth factor (HGF), vascular endothelial growth factor (VEGF), transforming growth factor beta (TGF-*β*), and interleukin- (IL-) 6) [3].

MSCs suppress immune response by attenuating migration and maturation of dendritic cells (DCs), promoting alternative activation of macrophages, and reducing proliferation, activation, and effector function of B and T lymphocytes, natural killer (NK), and natural killer T (NKT) cells [1].

Although proangiogenic and immunosuppressive characteristics of MSCs are beneficial in the treatment of degenerative and autoimmune diseases, they can represent a serious problem if patient that received MSCs have primary or metastatic tumor. Accordingly, safeness of MSC-based therapy is still a matter of debate. The primary concern was the potential malignant transformation of the administered MSCs. A recent meta-analysis partly reassured the scientific community by showing that malignancies which were noticed in MSC-treated patients occurred only in patients with previous or current malignancies, with no formation of de novo tumors [4]. However, potential of MSCs to promote neovascularization and suppress antitumor immunity is still existing as major concerns regarding the safety of MSC-based therapy and should be further explored in preclinical and clinical studies.

MSCs have at least three functions that can promote tumor metastasis: homing to the site where tissues are damaged, including metastatic lesions, production of immunomodulatory factors, and secretion of proangiogenic cytokines and growth factors that promote neovascularization enabling metastasis of tumor cells [1, 4].

Lung-infiltrated cells as well as their products which contribute to tumor escape mechanisms and host immunosuppression are emerging as important mediators in promoting lung cancer growth and metastasis [5, 6].

Since vast majority of intravenously injected MSCs initially become entrapped within the lungs where MSCs interact with tumor-infiltrated immune cells [1], we used metastatic model of murine lung cancer to investigate molecular and cellular mechanisms involved in MSC-mediated modulation of antitumor immune response and progression of lung cancer metastasis.

Herewith, we showed that intravenous application of MSCs in tumor-bearing mice significantly suppressed systemic antitumor immune response, reduced total number of lung-infiltrated DCs, macrophages, CD4+ T lymphocytes, CTLs, and NK cells, and attenuated antitumor cytotoxicity of CTLs and NK cells resulting with the expansion of metastatic lesions in the lungs.

## 2. Materials and Methods

### 2.1. Cells

MSCs isolated from bone marrow of C57BL/6 mice were purchased from Gibco (Catalog number S1502-100). The cells were cultured in complete Dulbecco's Modified Eagle Medium (DMEM) containing 10% heat-inactivated fetal bovine serum (FBS), 100 IU/mL penicillin G, and 100 *μ*g/mL streptomycin (Sigma-Aldrich, Munich, Germany), at 37°C in a 5% CO_2_ incubator. MSCs in passage 3 were used throughout the experiments.

The cell line of murine lung carcinoma (Lewis lung cancer 1, LLC1) (3LL, H2b), derived from the lungs of C57BL mice implanted with Lew (Lewis) lung cancer, was purchased from the American Type Culture Collection (ATCC) (Catalog number CRL-1642™). Cells were routinely grown in suspension in complete DMEM medium, at 37°C in a 5% CO_2_ incubator. LLC1 cells in passage 3 were used throughout the experiments.

### 2.2. Generation of MSC-Conditioned Medium (MSC-CM)

MSCs were seeded at a density of 10,000 cells/cm^2^. In order to collect the MSC-CM, MSCs were first cultured in serum-containing complete medium and incubated at 37°C in a humid atmosphere with 5% CO^2^. At 80% confluence, the cells were washed twice with 1X phosphate-buffered saline (PBS, Invitrogen), and the medium was then changed to serum-free medium. After 48 h, the medium was collected, centrifuged at 13000 ×*g* at 4°C for 10 min, and stored at −80°C until used [7].

### 2.3. Pharmacological Inhibition of Indoleamine 2,3-Dioxygenase (IDO) and Inducible Nitric Oxide Synthase (iNOS)

MSCs were cultured for 48 h in culture medium containing 1 mM 1-methyltryptophan, (1-MT, Sigma-Aldrich, St. Louis, MO), an inhibitor of IDO enzymatic activity [8].

To block iNOS activity, MSCs were cultured for 48 h in the presence of 1 mM of an iNOS inhibitor, L-N^G^-monomethyl arginine citrate (L-NMMA, Sigma-Aldrich, St. Louis, MO) [9].

### 2.4. Animals

6–8-week-old C57Bl/6 male mice were used. All animals received human care, and all experiments were approved by and conducted in accordance with the Guidelines of the Animal Ethics Committee of the Faculty of Medical Sciences of the University of Kragujevac, Serbia. Mice were housed in a temperature-controlled environment with a 12-hour light-dark cycle and were administered with standard laboratory chow and water ad libitum.

### 2.5. Induction of Experimental Metastasis and Systemic Application of MSCs

Experimental metastases were induced by intravenous injection of 5 × 10^4^ LLC1 cells [10]. Mice intravenously received either 5 × 10^5^ MSCs or saline one week after injection of LLC1 cells. Mice were sacrificed on the 28th day of the experiment, as previously described [5].

### 2.6. Histopathological Analysis

All mice were sacrificed in an atmosphere saturated with diethyl ether (BETA HEM, Belgrade), and the lungs were isolated for histopathological analysis of metastatic colonies 28 days after tumor induction.

The isolated lungs were fixed in 10% formalin and embedded in paraffin, and consecutive 4 *μ*m tissue sections mounted on slides. Sections were stained with hematoxylin and eosin (H&E) and examined under low-power (100x) light microscopy-equipped digital camera (Zeiss Axioskop 40, Jena, Germany). Metastases were verified by light microscopy (magnification 10x and 40x) as characteristic brown-black pigmented “hot spots” with giant multinucleated cells clearly limited by the surrounding lung tissue.

### 2.7. Immunohistochemistry

For immunohistochemical staining, paraffin-embedded sections (4 *μ*m) of mouse lung tissue were used. Heat-mediated antigen retrieval in citrate buffer (pH = 6.0) was performed. Deparaffinized tissue-sections were incubated with primary mouse anti-CD3 (sc-20047, Santa Cruz Biotechnology), anti-CD4 (ab183685, Abcam), anti-CD68 (ab49777, Abcam), anti-TNF-*α* antibody (ab6671, Abcam), and anti-IL-17 (ab79056, Abcam). Staining was visualized by using Mouse Specific HRP/DAB Detection IHC Kit (ab64259, Abcam) for CD3 and CD68, and rabbit specific HRP/AEC detection IHC Kit (ab94361, Abcam) for CD4, TNF-*α*, and IL-17. Sections were counterstained with Mayer's hematoxylin. Sections were photomicrographed with a digital camera mounted on light microscope (Olympus BX51, Japan), digitized, and analyzed.

### 2.8. Isolation of Lung-Infiltrated Immune Cells

The lungs, obtained from control, LLC1 and LLC1 + MSC-treated mice at the 28th day of the experiment, were washed with sterile phosphate-buffered saline (PBS) and placed in Petri dishes with DMEM supplemented with 10% FBS. The dissected lung tissue was incubated in medium that contained Collagenase Type IV (0.5 mg/mL) and type IV bovine pancreatic DNAse (Roche Diagnostic; 1 mg/mL) at 37°C for 45 min. The cells were filtered through a 100 *μ*m nylon cell strainer into a clean 50 mL conical tube. Then, cells were pelleted by centrifuging 10 min at 300 ×*g*, at 10°C. Red blood cells were depleted with a lysis buffer (0.144 M NH4Cl, 0.0169 M TRIS base, pH 7.4) at 37°C in a 5% CO_2_ atmosphere for 5 min [11].

### 2.9. Flow Cytometry Analysis and Intracellular Staining of Lung-Infiltrated Immune Cells

Lung-infiltrated immune cells were screened for various cell surface and intracellular markers with flow cytometry. Briefly, 1 × 10^6^ cells were incubated with anti-mouse CD45, F4/80, CD4, CD8, CD11c, CD11b, CD49b, FasL, CD107, perforin, NKG2D monoclonal antibodies conjugated with fluorescein isothiocyanate (FITC), phycoerythrin (PE), peridinin chlorophyll protein (PerCP), or allophycocyanin (APC) (all from BD Biosciences, San Jose, CA, USA) following the manufacturer's instructions. Immune cells derived from the lungs were concomitantly stained for the intracellular content of TNF-*α*, IL-10, and IL-17 by using the fixation/permeabilization kit and anti-mouse monoclonal antibodies conjugated with fluorescein isothiocyanate (FITC), phycoerythrin (PE), peridinin chlorophyll protein (PerCP), and allophycocyanin (APC) (BD Biosciences). For intracellular cytokine staining, cells were stimulated with 50 ng/mL PMA and 500 ng/mL ionomycin for 5 h, and GolgiStop (BD Biosciences) was added. Cells were fixed in Cytofix/Cytoperm, permeated with 0.1% saponin, and stained with fluorescent Abs. Flow cytometric analysis was conducted on a BD Biosciences' FACSCalibur and analyzed by using the flowing software analysis program.

### 2.10. Measurement of Cytokines and Growth Factors in Sera of Tumor-Bearing Mice

Levels of TNF-*α*, IL-17, IL-10, and HGF in the mouse serum at the 14th, 21st, and 28th days of the experiment were measured using ELISA kits specific for the mouse cytokines (R&D Systems, Minneapolis, MN, USA) according to the manufacturer's instructions.

Serum concentrations of nitric oxide (NO) were measured by Griess reagent while IDO activity was determined by spectrophotometric measuring of kynurenine since IDO catalyzes the metabolism of tryptophan in the kynurenine [12]. Concentrations of NO and kynurenine were determined in mouse sera at the 14th, 21st, and 28th days of the experiment.

### 2.11. Isolation of NK Cells by Magnetic Cell Sorting

At the 28th day of the experiment, NK cells were isolated from the spleens of LLC1 and LLC1 + MSC-treated mice by magnetic cell sorting according to the manufacturer's instructions. Single-cell suspensions of mononuclear cells derived from the spleens were labeled with monoclonal anti-mouse antibodies against CD49b, and microbeads conjugated to monoclonal anti-biotin antibody (Miltenyi Biotec). The labeled cells were subsequently depleted by separation over a MACS Column (Miltenyi Biotec), which was placed in the magnetic field of a MACS Separator (Miltenyi Biotec). Isolated NK cells were then used in the coculture experiments and cytotoxicity assay as purified NK cells.

### 2.12. Cytotoxicity Assay

The DP version of the xCELLigence system (Roche) was used in this study for the determination of NK cell cytotoxicity. The DP version comprises a measurement unit housed within a standard tissue culture incubator with 3 stations that each takes E16 plates (each E16 plate has 16 wells). 100 *μ*L of complete medium was added to each well, and background impedance on the plates was measured on the xCELLigence RTCA DP instrument at 37°C and 5% CO_2_. LLC1 cells were used as targets for NK cells. Seeding density of 4 × 10^4^ LLC1 cells/well was considered optimal and used for all assays. Effector to target ratio (E : T ratio) 10 : 1 was used [13]. LLC1 cells were resuspended in DMEM with 10% FCS at 4 × 10^5^ cells per milliliter. A total of 100 *μ*L tumor cells were added to each well of the E16 plate, which was then placed in the xCELLigence RTCA DP. NK cells, isolated from LLC1 and LLC1 + MSC-treated mice at the 28th day of the experiment, were counted and resuspended at a concentration of 4 × 10^6^ cells per milliliter in DMEM + 10% FCS media. Then, 100 *μ*L NK cells or media alone were added to the respective wells. The E-plate 16 was placed in the xCELLigence RTCA DP, and impedance measurements were recorded every 15 min for 24 hours at 37°C and 5% CO_2_. NK cell-mediated death of tumor cells was monitored in real time and was indicated by a decrease in cell index. Data were analyzed with RTCA Software 1.2 (ACEA Biosciences).

### 2.13. Statistical Analysis

The results were analyzed using the Student *t*-test. All data in this study were expressed as the mean ± standard error of the mean (SEM). Values of *p* < 0.05 were considered as statistically significant.

## 3. Results

### 3.1. Intravenous Injection of MSCs Significantly Augmented Lung Cancer Metastasis

**Figure 1:**
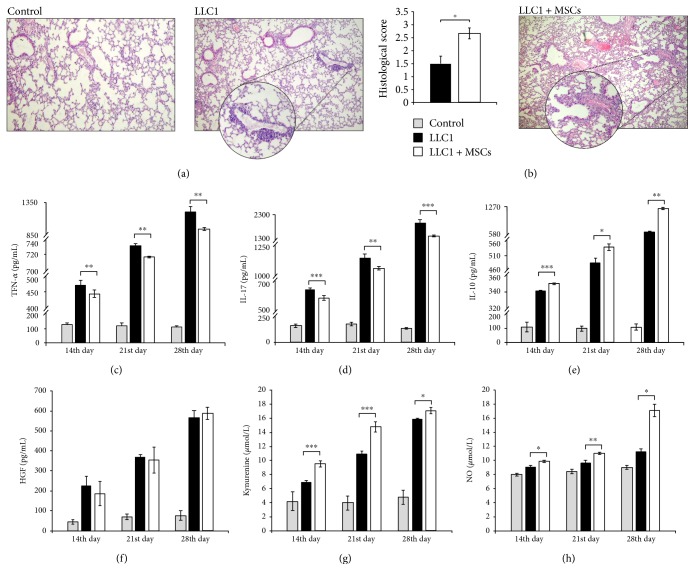
MSCs promote lung cancer metastasis. (a) Representative H&E stained mouse lungs obtained at the 28th day of the experiment. H&E staining images of liver tissue samples are shown at the same magnifications (×100). (b) Histological score of lung tissue determined at the 28th day of the experiment. (c) Serum concentrations of TNF-*α*, (d) IL-17, (e) IL-10, and (f) HGF measured at the 14th, 21st, and 28th days of the experiment. (g) The level of kynurenine and (h) NO in mouse sera at the 14th, 21st, and 28th days of the experiment. Data presented as mean ± SEM; *n* = 10 mice per experimental groups. ^∗^*p* < 0.05, ^∗∗^*p* < 0.01, and ^∗∗∗^*p* < 0.001.

First, we investigated whether systemic application of MSCs could modulate spontaneous LLC1 tumor cell metastasis to the lungs. We observed that LLC1 + MSC-treated tumor-bearing mice exhibited increased numbers of lung metastasis (Figure 1(a)) compared to animals that received only LLC1 cells. Significantly higher number of tumor cells with pleomorphic nuclei, arranged in aggregated forms, was noticed in the lungs of MSC-treated tumor-bearing animals at the 28th day of the experiment. Although perivascular infiltration of tumor cells was also noticed in the lungs isolated from LLC1-treated mice, expansion of malignant tissue in these mice was notably lower in comparison to LLC1 + MSCs-treated animals in which lung tissues were almost completely displaced with tumor cells (Figure 1(a)). Histological score of lung tissue confirmed extensive malignancy in LLC1-treated mice that intravenously received MSCs (Figure 1(b)).

### 3.2. MSCs Altered Serum Levels of Cytokine and Growth Factors That Played Important Role in Antitumor Immune Response

In order to explore whether MSC-dependent expansion of metastatic lesions in the lungs is a consequence of their effects on systemic immune response, cytokine concentration was determined in sera of tumor-bearing animals at the 14th, 21st, and 28th days of the experiment. In accordance with the histological analysis, MSCs significantly alter serum levels of cytokines and growth factors that play important role in antitumor immune response at all measured time points. The concentrations of antitumor cytokines TNF-*α* (Figure 1(c)) and IL-17 (Figure 1(d)) were significantly lower while the concentrations of immunosuppressive IL-10 (Figure 1(e)), kynurenine (Figure 1(g)), and NO (Figure 1(h)) were significantly higher in sera of LLC1-treated mice that received MSCs. There was not any significant difference in serum levels of immunomodulatory HGF between experimental groups (Figure 1(f)).

### 3.3. MSCs Significantly Reduced Total Number of DCs, Macrophages, and CD4+ Helper T Cells in the Lungs of LLC1-Treated Mice and Altered Their Cytokine Profile

**Figure 2:**
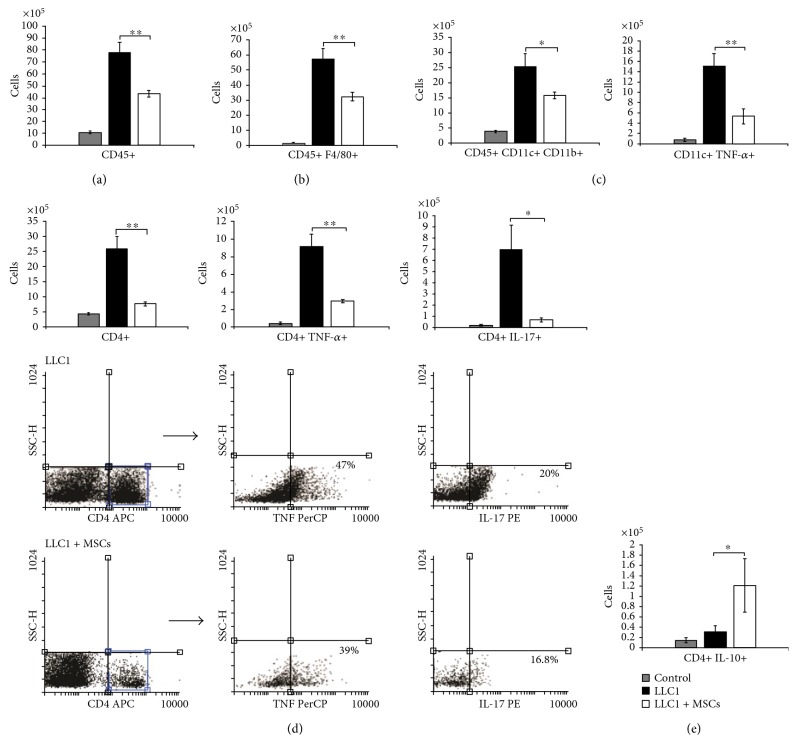
MSC treatment reduces influx of DCs, macrophages, and CD4+ T cells, in the metastatic model of lung cancer, and altered their cytokine profile. Total numbers of (a) CD45+, (b) CD45+ F4/80+, (c) CD45+ CD11c+ CD11b+, and CD11c+ TNF-*α*+ cells in the lungs of control, LLC1, and LLC1 + MSC-treated mice at the 28th day of the experiment. (d) Total number and representative flow cytometry dot plots of CD4+, TNF-*α*-, and IL-17-producing CD4+ cells at the 28th day of the experiment. (e) Total numbers of lung-infiltrating CD4+ IL-10+ cells at the 28th day of the experiment. Values are mean ± SEM (*n* = 10  per  group). ^∗^*p* < 0.05, ^∗∗^*p* < 0.01.

Next, we analyzed cellular make-up of the lungs 28 days after tumor injection in order to determine cellular targets of MSC-mediated suppression of antitumor immune response in LLC1-treated animals. MSCs profoundly reduced infiltration of CD45+ leukocytes into the lung parenchyma (*p* < 0.01; Figure 2(a)). Flow cytometry analysis showed that a total number of CD45+ F4/80+ macrophages (Figure 2(b), *p* < 0.01), CD45+ CD11c+ CD11b+ inflammatory DCs (Figure 2(c), left panel, *p* < 0.05), and CD4+ helper T cells (Figure 2(d), *p* < 0.01) were significantly lower in the lungs of tumor-bearing mice that received MSCs.

Intracellular staining revealed that systemic application of MSCs reduces infiltration of TNF-*α*-producing DCs (Figure 2(c), right panel, *p* < 0.05), TNF-*α*-producing (Figure 2(d), middle panel, *p* < 0.01), and IL-17-producing CD4+ helper T cells (Figure 2(d), right panel, *p* < 0.05) and increases the presence of CD4+ T cells that produce immunosuppressive IL-10 (Figure 2(e), *p* < 0.05).

**Figure 3:**
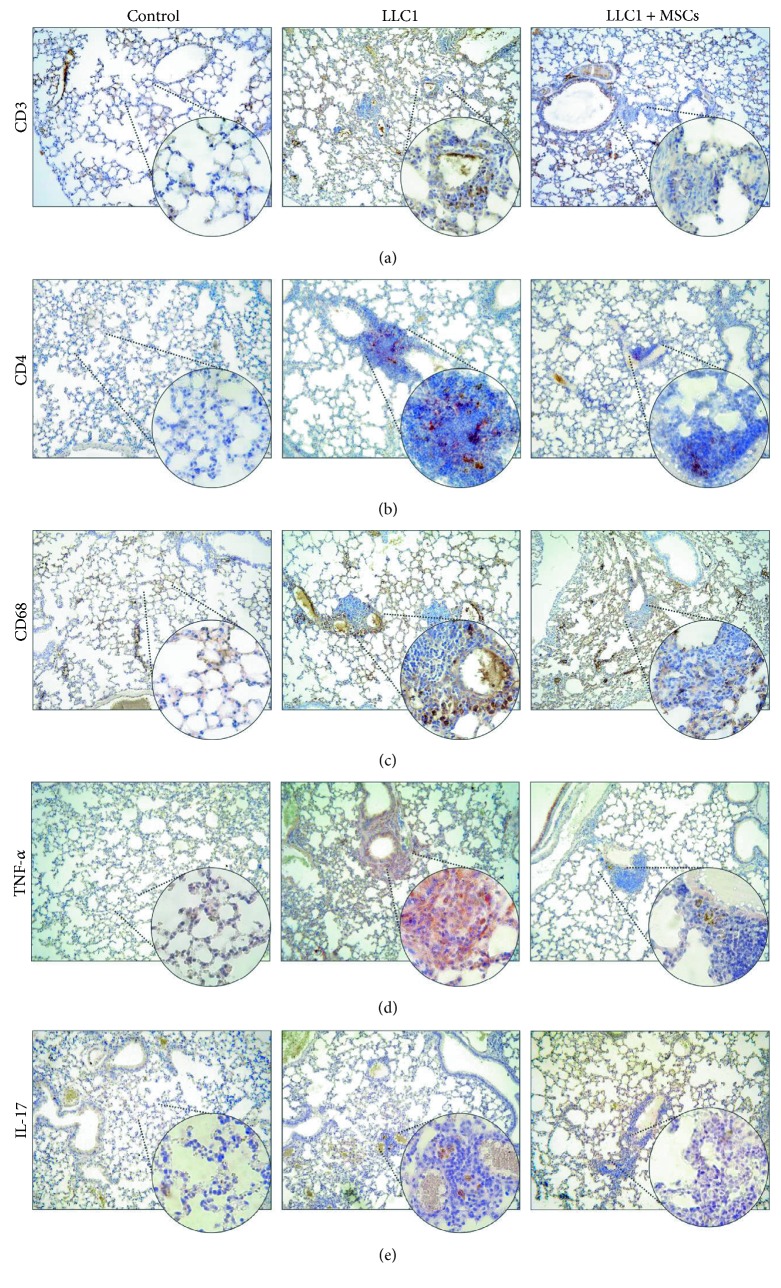
Systemic injection of MSCs reduces the presence of immune cells and inflammatory cytokines in the lungs of LLC1-treated mice. Representative images of CD3, CD4, CD68, TNF-*α*, and IL-17 immunohistochemical staining on paraffin-embedded lung tissue sections obtained at the 28th day of the experiment (×20, ×40). (a) CD3+ cells, (b) CD4+ cells, and (c) CD68+ macrophages were present in higher numbers in lungs of LLC1-treated mice compared to LLC1 + MSCs and control groups. Expression of (d) TNF-*α* and (e) IL-17 in lung tissue was higher in lungs of LLC1-treated mice compared to LLC1 + MSC and control groups.

Immunohistochemical analysis confirmed these findings. Intravenous injection of MSCs reduced the presence of CD3+ T lymphocytes (Figure 3(a)), CD4+ T helper cells (Figure 3(b)), CD68+ macrophages (Figure 3(c)), TNF-*α*-producing (Figure 3(d)), and IL-17-producing cells (Figure 3(e)) in the lungs of LLC1-treated mice 28 days after tumor induction.

### 3.4. MSCs Reduced Infiltration of CTLs in the Lungs of LLC1-Treated Mice and Attenuated Expression of FasL, Perforin, and CD107 on Their Surface

**Figure 4:**
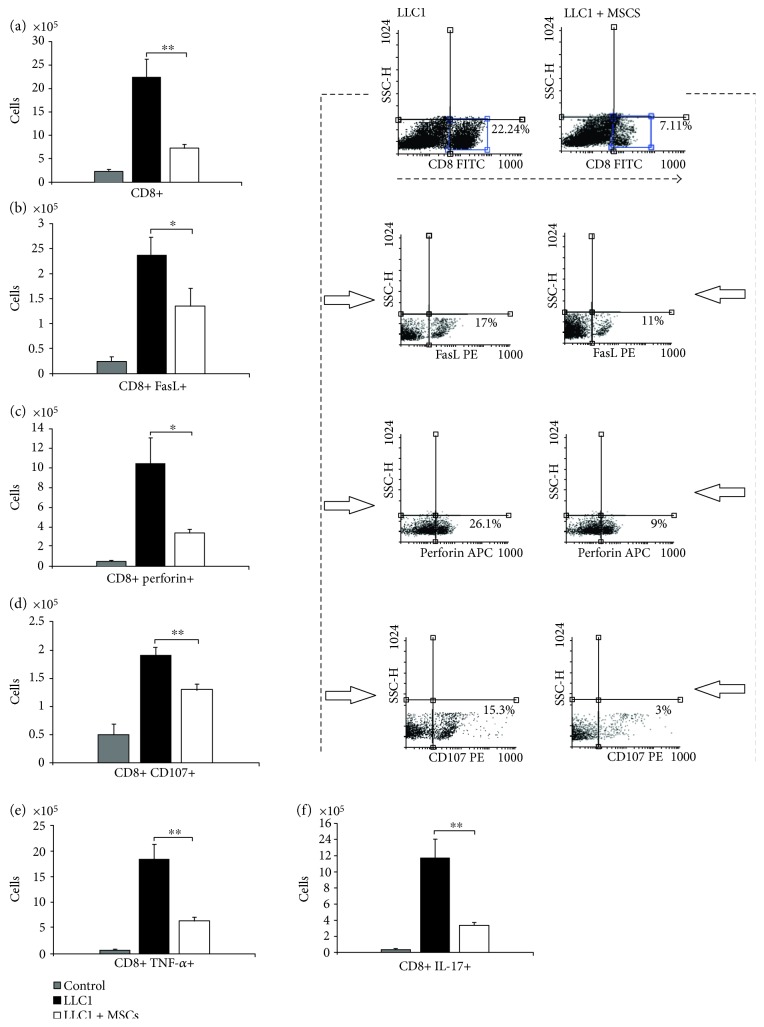
MSCs reduce infiltration of CTLs in the lungs of LLC1-treated mice and attenuate expression of FasL, perforin, and CD107 on their surface. Total number of (a) CD8+, (b) CD8+ FasL+, (c) CD8+ perforin+, (d) CD8+ CD107+, (e) CD8+ TNF-*α*+, and (f) CD8+ IL-17+ cells in the lungs of control, LLC1, and LLC1 + MSC-treated mice at the 28th day of the experiment, accompanied with representative dot plots. Data presented as mean ± SEM; *n* = 10 mice per experimental groups. ^∗^*p* < 0.05, ^∗∗^*p* < 0.01.

Intravenous injection of MSCs significantly reduce total number of cytotoxic CD8+ CTLs (Figure 4(a), *p* < 0.01) in the lungs of LLC1-treated mice at the 28th day of the experiment. Moreover, analysis of cytotoxic molecules involved in CTL-mediated antitumor immune response (FasL, perforin, and CD107) showed that MSCs managed to attenuate influx of FasL+ CTLs (Figure 4(b), *p* < 0.05), perforin + CTLs (Figure 4(c), *p* < 0.05), and CD107+ CTLs (Figure 4(d), *p* < 0.01) in the lungs of tumor-bearing animals.

Intracellular staining revealed that systemic application of MSCs attenuate the capacity of CTLs to produce antitumor cytokines (Figures 4(e)-4(f)). There were significantly lower number of TNF-*α*-producing (Figure 4(e), *p* < 0.01) and IL-17-producing CTLs (Figure 4(f), *p* < 0.01) in MSC + LLC1-treated mice when compared to LLC1-only treated animals.

### 3.5. Antitumor Cytotoxicity of NK Cells Was Significantly Attenuated in MSC + LLC1-Treated Mice in Paracrine, NO, and IDO-Dependent Manner

**Figure 5:**
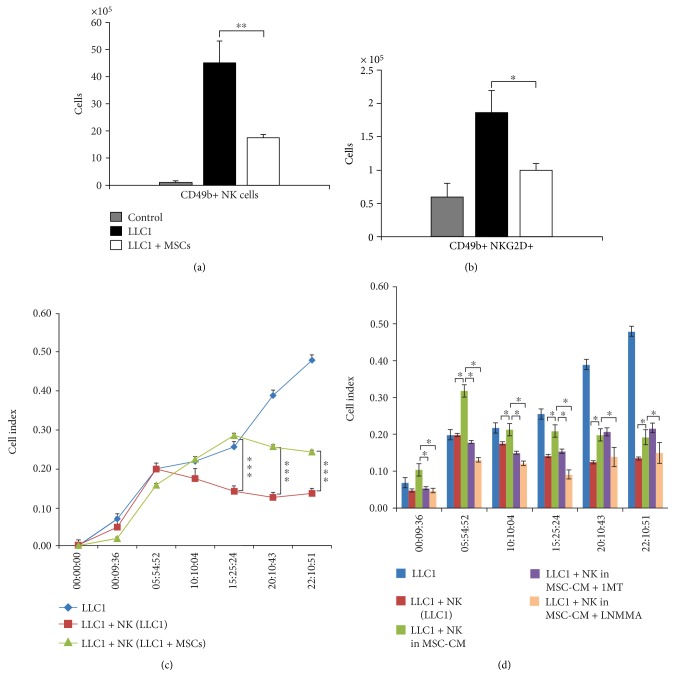
MSCs attenuate antitumor cytotoxicity of NK cells in iNOS and IDO-dependent manner. The total number of (a) CD49b+ and (b) CD49b+ NKG2D+ cells in the lungs of control, LLC1, and LLC1 + MSC-treated mice at the 28th day of the experiment. (c-d). The results obtained by xCELLigence system showed cytotoxic activity of NK cells against LLC1 target cells. Data presented as mean ± SEM; *n* = 10 mice per experimental groups. ^∗^*p* < 0.05, ^∗∗^*p* < 0.01, and ^∗∗∗^*p* < 0.001.

As determined by flow cytometry, 28 days after tumor induction, the presence of CD49b + NK cells was significantly reduced in LLC1 + MSC-treated mice when compared to the LLC1-only treated animals (Figure 5(a), *p* < 0.01).

Moreover, the total number of NK cells that express activation receptor NKG2D, involved in antitumor immune response, was notably lower in the lungs of tumor-bearing mice that received MSCs (Figure 5(b), *p* < 0.05). Accordingly, the results obtained by xCELLigence system for monitoring real-time cytotoxicity showed that NK cells isolated from LLC1 + MSC-treated mice were significantly less cytotoxic against LLC1 cells then NK cells isolated from animals that received only LLC1 cells (Figure 5(c)), indicating that intravenous injection of MSCs significantly reduced antitumor cytotoxicity of NK cells.

To directly demonstrate that soluble factors and not cell to cell contact were responsible for the MSC-mediated inhibition of NK cell cytotoxicity against LLC1 cells, effects of MSC-CM were evaluated. As it is shown in Figure 5(d), MSC-CM significantly suppressed cytotoxicity of NK cells against LLC1 cells. This phenomenon was completely abrogated in the presence of iNOS inhibitor (L-NMMA) or IDO inhibitor (1-MT), suggesting iNOS and IDO as important factors for MSC-mediated suppression of antitumor cytotoxicity of NK cells (Figure 5(d)).

## 4. Discussion

It is well known that MSCs are inherently tumor-homing cells that, few hours after systemic injection, migrate in the lungs [14, 15]. Many receptors, extracellular matrix proteins, and soluble tumor-derived factors have been reported to effect tumor tropism of intravenously injected MSCs [15, 16]. Most recently, it was shown that macrophage migration inhibitory factor (MIF)/CXCR4 and monocyte chemoattractant protein-1 (MCP-1)/CCR2 pathways are responsible for migration of MSCs in the tumor microenvironment of the lungs [15, 16]. MIF secreted from tumor cells attracts MSCs to the lungs in CXCR4-dependent manner. Knockdown of either CXCR4 or MIF abrogates MSC homing to tumors in an in vivo pulmonary metastasis model [15]. Similarly, homing ability of MSCs was suppressed after either knocking down the expression of MCP-1 in lung cancer cells or blocking CCR2 expressed on the surface of MSCs, indicating the important role of MCP-1/CCR2 axis in the tropism of MSCs to lung tumors [16]. Immediately after their engraftment, MSCs interact with tumor-infiltrated immune cells, affecting antitumor immune response [17]. Accordingly, findings related to the MSC-based modulation of antitumor immunity could have important implications for clinical applications of MSCs.

Here, we provide the evidence that systemic application of MSCs in tumorbearing animals promotes expansion of lung metastasis by suppressing antitumor immune response through the inhibition of innate (DCs, NK cells) and adaptive (CD4+ T helper, CD8 + CTLs) immunity.

Significantly lower number of macrophages (Figures 2(b) and 3(c)), DCs (Figure 2(c)), effector CD4+ helper T cells (Figures 2(d) and 3(b)), and CD8+ CTLs (Figures 4(a), 4(b), 4(c), 4(d), 4(e), and 4(f)) as well as reduced number and cytotoxicity of NK cells (Figure 5) indicates that systemic application of MSCs affected both inductive and effector phase of antitumor immune response.

Intravenous injection of MSCs suppresses, almost instantaneously, the migration of DCs to the draining lymph nodes significantly affecting the ability of DCs for antigen presentation to CD4+ T cells and cross-presentation to CD8+ T cells [18, 19]. Accordingly, significantly reduced number of CD11c+ CD11b+ DCs (Figure 2(c)) was accompanied with reduced number of CD4+ (Figures 2(d) and 3(b)) and CD8+ T cells (Figure 3(a)) in the lungs of LLC1-treated mice that received MSCs.

Maturation of DC is also impaired by MSCs [20, 21]. DC exposed to TNF-alpha and MSCs failed to upregulate maturation markers [19]. On turn, immature DCs are strongly hampered in their ability to produce TNF-*α* and other proinflammatory cytokines that inhibit tumor growth and metastasis [22]. In line with these observations, downregulated serum levels of TNF-*α* (Figure 1(c)) were accompanied with lower number of TNF-*α*-producing DCs in the lungs of LLC1 + MSCs-treated mice when compared to LLC1-only treated animals (Figure 2(c)).

In addition to suppression of naïve T cell activation, MSCs are able to induce suppression of effector CD4+ T helper cells that is mainly mediated through the production of soluble factors including IDO, NO, IL-10, prostaglandin E2 (PGE2), HGF, and TGF-*β* [23]. Accordingly, intravenous injection of MSCs resulted with higher serum levels of IL-10 (Figure 1(e)), kynurenine (Figure 1(g)), and NO (Figure 1(h)) that was followed by reduce number of lung-infiltrated effector CD4+ T cells (Figures 2(d) and 3(b)) that produce antitumor cytokines TNF-*α* and IL-17 (Figure 2(d)).

Progressive inflammatory diseases, including tumors, are associated with the loss of IL-17-producing CD4+ Th17 cells and a reciprocal increase in the fraction of the immunosuppressive IL-10-producing CD4+ T lymphocytes both in peripheral blood and in inflamed tissues [24]. MSC-derived IDO is an enzyme that has powerful immunomodulatory effects, resulting from its enzymatic activity, which leads to catabolism of the essential aminoacid L-tryptophan to L-kynurenine [25]. Metabolites of the L-kynurenine pathway have been shown to act as critical molecular switch that stimulates immunosuppressive properties of IL-10producing T cells and simultaneously blocks their reprogramming into IL-17-producing effector T cells [26]. Accordingly, herewith, we showed that injection of MSCs increased serum levels of kynurenine in LLC1-treated mice (Figure 1(g)), accompanied with increased number of IL-10-producing CD4+ T lymphocytes and decreased number of IL-17-producing Th17 cells (Figure 2(d)).

As described above, MSCs attenuate the capacity of DC for cross-presentation and activation of CD8+ T cells [18]. Additionally, MSCs suppress the proliferation of CTLs and inhibit surface expression of molecules which are involved in CTL-mediated cytotoxicity against tumor cells [27]. In line with these findings, significantly lower number of lung-infiltrated CD8 + CTLs expressing FasL (Figure 4()(b)) and perforin (Figure 4()(c)) was noticed in MSC + LLC1-treated mice when compared to LLC1-only treated animals indicating that systemic administration of MSCs suppressed both perforin- and FasL-mediated mechanisms of antitumor cytotoxicity of CTLs.

MSCs suppress proliferation and cytotoxicity of NK cells, as well [28–30]. This inhibitory effect of MSCs is associated with the downregulated expression of the activating NK cell receptors, such as NKG2D, and is primarily mediated by IDO, PGE2, and TGF-*β*1 [29, 30]. Herewith, we showed that MSCs reduce total number of lung-infiltrated NKG2D-expressing NK cells in LLC1-treated mice (Figure 5(b)) and significantly attenuate their cytotoxicity against lung cancer cells in vitro (Figure 5(c)). Both iNOS and IDO inhibitors managed to almost completely restore cytotoxic activity of NK cells against LLC1 cells in vitro (Figure 5(d)), suggesting the importance of both iNOS and IDO signaling for MSC-mediated inhibition of NK cell antitumor toxicity. It is well known that inflammatory cytokines, such as TNF-*α*, provoke MSCs to use iNOS-dependent mechanism for NO production [9, 31]. MSC-derived NO can directly suppress proliferation of lymphocytes or may increase IDO activity which could result with the attenuation of NK cell cytotoxicity [9, 31].

## 5. Conclusions

Our study provides the evidence that systemic application of MSCs may promote metastasis of lung cancer cells by suppressing antitumor immune response. These findings raised serious concerns regarding the safety of intravenous application of MSCs in patients who have genetic susceptibility for malignant diseases.
